# Lipocalin-2 promotes adipose–macrophage interactions to shape peripheral and central inflammatory responses in experimental autoimmune encephalomyelitis

**DOI:** 10.1016/j.molmet.2023.101783

**Published:** 2023-07-28

**Authors:** Francesca Sciarretta, Veronica Ceci, Marta Tiberi, Fabio Zaccaria, Haoyun Li, Zhong-Yan Zhou, Qiyang Sun, Daniels Konja, Alessandro Matteocci, Anup Bhusal, Martina Verri, Diego Fresegna, Sara Balletta, Andrea Ninni, Claudia Di Biagio, Marco Rosina, Kyoungho Suk, Diego Centonze, Yu Wang, Valerio Chiurchiù, Katia Aquilano, Daniele Lettieri-Barbato

**Affiliations:** 1 IRCCS, Fondazione Santa Lucia, 00179 Rome, Italy; 2 PhD Program in Evolutionary Biology and Ecology, Department of Biology, University of Rome Tor Vergata, 00133 Rome, Italy; 3 Department of Biology, University of Rome Tor Vergata, 00133 Rome, Italy; 4 Laboratory of Resolution of Neuroinflammation, IRCCS Santa Lucia Foundation, 00179 Rome, Italy; 5 The State Key Laboratory of Pharmaceutical Biotechnology; 6 Department of Pharmacology and Pharmacy, The University of Hong Kong, Hong Kong SAR, China; 7 Longhua Hospital, Shanghai University of Traditional Chinese Medicine, Shanghai, China; 8 PhD program in Immunology, Molecular Medicine and Applied biotechnologies, University of Rome Tor Vergata, 00133 Rome, Italy; 9 Department of Pharmacology, School of Medicine, Kyungpook National University, Daegu 41944, Republic of Korea; 10 BK21 Plus KNU Biomedical Convergence Program, Department of Biomedical Science, School of Medicine, Kyungpook National University, Daegu 41944, Republic of Korea; 11 Synaptic Immunopathology Lab, IRCCS San Raffaele Pisana, 00163 Rome, Italy; 12 Department of Systems Medicine, Tor Vergata University, 00133 Rome, Italy; 13 Unit of Neurology, IRCCS Neuromed, 86077 Pozzilli, Italy; 14 Neurology Unit, Fondazione PTV Policlinico Tor Vergata, Viale Oxford 81, 00133 Rome, Italy; 15 Brain Science and Engineering Institute, Kyungpook National University, Daegu 41944, Republic of Korea; 16 Institute of Translational Pharmacology, National Research Council, 00133 Rome, Italy; 17 Pathology Unit, University Hospital Campus Bio-Medico of Rome, 00128 Rome, Italy

**Keywords:** Adipose tissue, Immune cells, Lipid metabolism, Mitochondria, Macrophages, Adipocyte, Cachexia

## Abstract

**Objective:**

Accumulating evidence suggests that dysfunctional adipose tissue (AT) plays a major role in the risk of developing multiple sclerosis (MS), the most common immune-mediated and demyelinating disease of the central nervous system. However, the contribution of adipose tissue to the etiology and progression of MS is still obscure. This study aimed at deciphering the responses of AT in experimental autoimmune encephalomyelitis (EAE), the best characterized animal model of MS.

**Results and Methods:**

We observed a significant AT loss in EAE mice at the onset of disease, with a significant infiltration of M1-like macrophages and fibrosis in the AT, resembling a cachectic phenotype. Through an integrative and multilayered approach, we identified lipocalin2 (LCN2) as the key molecule released by dysfunctional adipocytes through redox-dependent mechanism. Adipose-derived LCN2 shapes the pro-inflammatory macrophage phenotype, and the genetic deficiency of LCN2 specifically in AT reduced weight loss as well as inflammatory macrophage infiltration in spinal cord in EAE mice. Mature adipocytes downregulating LCN2 reduced lipolytic response to inflammatory stimuli (e.g. TNFα) through an ATGL-mediated mechanism.

**Conclusions:**

Overall data highlighted a role LCN2 in exacerbating inflammatory phenotype in EAE model, suggesting a pathogenic role of dysfunctional AT in MS.

## Highlights

• EAE causes a massive WAT loss with inflammatory cell infiltration and fibrosis.
• Dysfunctional AT release LCN2 through a redox-dependent mechanism.
• AT-derived LCN2 promotes macrophage activation.
• LCN2 downregulation protects against demyelination e inflammation in EAE.
• Adipocytes downregulating LCN2 limits weight loss, reduced lipolysis and myelin sheath inflammation in EAE mice.

## 1 Introduction

Multiple Sclerosis (MS) is an immune-mediated process in which an abnormal response of the body's immune system is directed against myelin sheath [1]. Although the etiology of MS is still unknown, a tight relationship between dysfunctional adipose tissue and MS has been observed [2]. Adipose tissue (AT) is a fat-rich tissue and is characterized by a complex cell-to-cell network involving immune cells that respond to environmental stimuli [3]. Consistent with the role of AT dysfunction in MS, several authors observed that experimental autoimmune encephalomyelitis (EAE), the most commonly used experimental murine model for the study of MS, induces ∼15–20% body weight loss (mainly due to the loss of adipose mass), at the disease onset [[4], [5], [6], [7]]. Similar results were obtained in cuprizone-treated mice, a well-established model to study demyelination in rodents [[8], [9], [10], [11]]. Remarkably, a massive weight loss was also described in EAE mice fed with high fat diet [12]. Even if many efforts have been made in the last years to identify a pathophysiological role of AT in MS, to date scarce evidence is available about the role of AT in the pathogenesis of MS. Dissecting the immunogenic potential of AT in murine models of MS could shed light on new pathogenetic mechanisms of this disease.

Corroborating studies have also revealed that adipocytes release a plethora of secretory factors perturbing the immuno-metabolic homeostasis and could have a role in autoimmune diseases, including MS [[13], [14], [15], [16]]. Among these, a role for lipocalin 2 (LCN2) in the pathogenesis of MS is emerging [[17], [18], [19], [20]]. LCN2 is a siderophore-binding protein mainly released from AT (also referred as adipokine) [16,17,21]. LCN2 is increased in several pathological settings including MS and cancer-associated cachexia [21,22]. LCN2 directly promotes muscle atrophy, activation of immune cells and sarcopenia in obese mice [23,24]. Mice overexpressing LNC2 specifically in the adipose tissue (Tg-Adipose-LCN2) showed reduced body weight, which was accompanied by lower AT mass if compared with WT mice. Furthermore, Tg-Adipose-LCN2 also increased the energy dissipating phenotype in the adipose depots [25]. Additionally, mice with global LCN2 genetic ablation (Lcn2KO) led to accelerated weight gain and visceral fat deposition with age, when compared to wild type (WT) mice [26]. In MS patients, the increased levels of LCN2 seems to contribute to neurodegeneration through myelination-dependent pathway [17,20]. In murine models of MS, total ablation of Lcn2 (Lcn2 KO) ameliorates EAE-related symptoms, suggesting that LCN2 expression in spinal cord and peripheral immune organs contribute to EAE development [18]. In EAE models, recent findings described LCN2 up-regulation in MS lesions at the single-cell level {Fournier, 2023 #53). Although AT results as the greater sink of circulating LCN2 in the body, a direct role of AT-derived LCN2 in modulating inflammation in EAE mice are unexplored.

Herein we aimed at exploring the AT responses at the onset of EAE and we identified LCN2 as main protein released from dysfunctional adipocytes able to exacerbates the inflammatory setting in a mouse model of MS.

## 2 Materials and methods

### 2.1 Mouse models and treatments

Experimental Autoimmune Encephalomyelitis (EAE) was induced in female C57BL/6 mice (6–8 weeks old) purchased from Charles River as previously described. Briefly, mice were injected subcutaneously with 200 μL of emulsion containing 200 μg of myelin oligodendrocyte glycoprotein 35–55 (MOG_35–55_) in Complete Freund's Adjuvant (CFA) containing 5 mg/mL of *Mycobacterium tuberculosis* (H37Ra strain, Difco), followed by intravenous administration of pertussis toxin (500 ng) twice (at days 0 and 2) as previously described ([27]{Leuti, 2021 #55). Control animals received the same treatment as EAE mice without the immunogen MOG peptide (CFA). The animals were scored daily for clinical symptoms of EAE, according to the following scale: 0 = healthy; 1 = flaccid tail; 2 = ataxia and/or paresis of hindlimbs; 3 = paralysis of hindlimbs and/or paresis of forelimbs; 4 = tetraparalysis; 5 = moribund or death due to EAE. Intermediate clinical signs were scored adding 0.5 value. In EAE mice, first clinical symptoms (disease onset) appeared about 12–16 days post immunization (dpi) with a peak of severity at about 19–22 dpi. This stage is referred to as symptomatic or acute phase of the disease. Spleens and cells were extracted during this stage. C57BL/6 mice (Charles-River, Sulzfeld, Italy) were randomly assigned to standard cages (4–5 animals per cage) and kept at standard housing conditions with a light/dark cycle of 12 h and free access to food and water. Beginning one week before the immunization, all animals were kindly handled every day to reduce the stress induced by operator manipulation during behavioral experiments.

Cancer-associated cachexia was induced in female C57BL/6 mice (6–8 weeks old) purchased from Charles River. C57BL/6 male mice (3 m. o.) were subcutaneously inoculated in the right flank with 5 × 10^6^ Lewis Lung Carcinoma (LLC) cells in a total volume of 100 μl of sterile PBS. Mice were maintained for 21 days under standard housing conditions and the total body weight, and the volume of tumor mass was monitored every other day. The tumor mass was measured with a caliber and the volume was calculated through formula *V* = *a∗(bˆ2/2)* where *a* is the major diameter and *b* is the minor diameter, according to [28]. The cachectic index, expressed as percentage of body weight loss, was calculated through the formula *C.I.* = *[(iWt – fWt + fTW + cWI)∗100]/(iWt + cWI)*, where *C.I.* = cachectic index; *iWt* = initial weight of treated animal; *fWt* = final weight of treated animal; *fTW* = final tumor weight; *cWI* = mean control animals weight increase, according to [29]. Mice were euthanized through cervical dislocation at 21 d. p.i. or when C.I. reached 15%, to reduce animal sufferance. Animal experiments were performed according to the Internal Institutional Review Committee, the European Directive 2010/63/EU and the European Recommendations 526/2007, and the Italian D. Lgs 26/2014.

Global *Lcn2* KO C57BL/6 mice were provided by Dr. Shizuo Akira from Osaka University, Japan. These mice were then backcrossed for 8–10 generations with a C57BL/6 strain to create homozygous animals [30]. The genomic DNA was extracted and PCR was performed to verify the absence of *Lcn2* gene in these animals. EAE was induced in global *Lcn2* KO mice as described above in mouse model section.

Adipo Lcn2 KO mice were generated was previously described [31]. For all studies, the Lcn2-floxed (Flox) littermates served as controls in experiments on AdipoLKO.

### 2.2 Cell culture and treatments

3T3-L1 cells were purchased from the American Type Culture Collection (ATCC,USA). 3T3-L1 pre-adipocytes were cultured in Dulbecco's Modified Essential medium (DMEM, Gibco, USA), supplemented with 10% of bovine calf serum (Euroclone), 100 U/ml penicillin, 10 mg/mL streptomycin (1% P/S) (Life technologies, Carisbad CA, USA) at 37 °C in a humidified incubator containing 5% of CO_2_. For differentiation induction, 10000 cells/cm^2^ were seeded and maintained at 100% of confluence for 48 h. Then cells were cultured with DMEM supplemented with 10% of heat inactivated Fetal Bovine Serum (FBS, Euroclone), 100 U/ml penicillin, 10 mg/mL streptomycin (1% P/S) (Life technologies, Carisbad CA, USA), 1 ug/ml insulin (Sigma-Aldrich, Saint Louis, MO, USA), 1 uM Dexamethasone (DEXA, Sigma-Aldrich, Saint Louis, MO, USA), 500 uM 3-Isobutyl-1-methylxanthine (IBMX, Sigma-Aldrich, Saint Louis, MO, USA). After 48 h of induction, fresh medium containing only insulin 1 ug/ml (Sigma-Aldrich, Saint Louis, MO, USA) was added every 2 days up to 8 days. For inflammation induction differentiated 3T3-L1 cells were treated with Tumor Necrosis Factor (TNF-α, Sigma-Aldrich, Saint Louis, MO, USA) 10 ng/mL for 24 h. To induce mitochondrial dysfunction, differentiated 3T3-L1 cells were treated with Cobalt Chloride (CoCl_2_, Sigma-Aldrich, Saint Louis, MO, USA) 100 μM, and Antimycin (AA, Sigma-Aldrich, Saint Louis, MO, USA) 1 μM for 16 h. To study the protective effect of Dimethyl fumarate (DMF) against the TNF-α, AA or CoCl_2_ induced damage, differentiated 3T3-L1 cells were pre-treated with 100 μM of DMF (Sigma-Aldrich, Saint Louis, MO, USA) for 1 h and then treated with TNF-α, AA or CoCl_2_, maintaining the DMF treatment. For the study of anti-REDOX mechanism, 3T3-L1 were pre-treated with N-acetylcysteine (NAC, Sigma-Aldrich, Saint Louis, MO, USA) 3 mM for 2 h and then treated with TNF-α, AA or CoCl_2_ maintaining the NAC treatment. For ATGL inhibition, differentiated 3T3-L1 cells were pre-treated with ATGL-istatin (ATGL_i, Sigma-Aldrich, Saint Louis, MO, USA) 200 uM for 1 h and then treated with TNF-α, maintaining the ATGL_i treatment.

For the lipolysis inhibition differentiated 3T3-L1 cells were pre-treated with H89 10 μM (Sigma-Aldrich, Saint Louis, MO, USA) for 1 h and then treated with TNF-α 10 ng/mL and IBMX 500 μM (Sigma-Aldrich, Saint Louis, MO, USA) maintaining the H89 treatment. All the treatments were done for 16 h and in DMEM serum free. For LCN2 gene silencing, differentiated 3T3-L1 cells were transfected with 40 pmol of small interfering RNA (LCN2 or scramble sequence, Origene) using Lipofectamine ™ 2000 transfection reagent, according to manufacturer's instructions (ThermoFisher Waltarm, MA, USA). After 48 h from transfection, cells were treated with TNF-α (Sigma-Aldrich, Saint Louis, MO, USA) 10 ng/mL for 16 h.

Firstly, the mixed glia culture was prepared using the brains of 3-day-old mice as described previously [32]. The acquired mixed glial cells were seeded in culture flasks and grown in an incubator at 37 °C with 5% CO2 in Dulbecco's Modified Eagle's medium (DMEM) (Hyclone Laboratories, South Logan, Utah) supplemented with 10% FBS and 100 U/ml of penicillin, and 100 μg/mL of streptomycin (Gibco, Grand Island, NY). Culture media was changed initially on day 5 and then every 3 days. Following 14 days of culture, primary microglia were obtained from mixed glial cells using a mild trypsinization method and maintained in DMEM [33]. The purity of primary microglia cultures was greater than 95%, as identified by Iba-1 staining [34].

RAW 264.7 cells were purchased from the American Type Culture Collection (ATCC, USA). RAW 264.7 were cultured in DMEM, supplemented with 10% FBS, 100 U/ml penicillin, 10 mg/mL streptomycin (1% P/S) (Life technologies, Carisbad CA, USA) at 37 °C in a humidified incubator containing 5% of CO_2._

For co-culture experiments, after the transfection with LCN2 (Origene, cat no. SR405112) or LCN2 receptor (Slc22a17; Origene, cat no. SR410149) siRNA and the TNF-α treatment, 3T3L-1 were co-cultured with RAW 264.7 for 24 h using transwell (Falcon) with 0.4 μm pores.

### 2.3 Lipolysis detection assay

Media were collected after the treatments, centrifuged at 400 g for 5 min to remove dead cell and debris, and used for glycerol quantification. Glycerol assay kit (ThermoFisher Waltarm, MA, USA) was used according with manufacturer's instructions.

### 2.4 Nucleus and cytoplasm fractionation

For nucleus and cytoplasm extraction, 3T3-L1 cells were lysed in 1 mL of extraction buffer (EB; Hepes 1 mM, 0.5% NP-40, KCl 10 mM, MgCl 1.5 mM, Sucrose 250 mM and protease and phosphatase inhibitors from Sigma-Aldrich) and incubated in ice for 15 min, then centrifuged at 1500 g for 5 min at 4 °C. Supernatant (cytosolic faction) was collected and pellet (nuclei) were washed twice with 1 mL of EB and centrifuged at 1500 g for 5 min at 4 °C. Nuclei were resuspended with 200 μl of TBS buffer (NaCl 137 mM, KCl 2.7 mM, Tris-base 24,7 mM, 0.1% SDS). After protein dosage with Lowry protocol, protein where denaturized and loaded on polyacrylamide gels.

### 2.5 Immunoblotting

Tissue samples and Cells were lysed in RIPA buffer (Tris-base 50 mM, NaCl 150 mM, 1% of NP-40, 0.5% of deoxycholic acid, NaF 1 mM, Sodium orthovanadate 1 mM, and protease and phosphatase inhibitors from Sigma-Aldrich). After protein dosage with Lowry protocol, protein where denaturized adding Sample Buffer (Tris base 125 mM, 4% of Sodium dodecyl sulphate, 20% of Glycerol, 0.004% of bromophenol blue, 10% of beta-mercapto-ethanol) at 1:1 ratio and then denaturated the samples at 96 °C for 5 min. Then 15 μg of proteins were loaded on polyacrylamide gels (SDS-PAGE) and then subjected to Immunoblotting. Nitrocellulose membranes were incubated with anti-LCN2 (Abcam), anti-αSMA (Abcam), anti-HSL (Cell signaling), anti-pHSL660 (Cell signaling), anti-PPARγ (Santa Cruz), anti-Vinculin (Invitrogen), anti-NFR2 (Sigma Aldrich, Saint Louis, MO, USA), anti-LDH (Abcam), anti-ATGL (Cell signaling) primary antibodies at 1:1000 dilution. Membranes were then incubated with Horseradish peroxidase-conjugated secondary antibodies. For protein detection, membranes were incubated with ECL Selected Western Blotting Detection Reagent (Biorad) and then immunoreactive bands were detected using a Fluorchem FC3 System (Protein-simple, San Jose, CA, USA). Densitometric analysis of the immune reactive bands were performed using Imagej analysis Software.

### 2.6 Bulk RNA-sequencing and data integration

The adipose tissue samples were subject to RNA-sequencing using an Illumina NextSeq550 and the indexed libraries were prepared from 1 μg-purified RNA with TruSeq-stranded mRNA (Illumina) Library Prep Kit according to the manufacturer's instructions. The quality of the single-end reads was evaluated with FastQC v.0.11.5 (https://www.bioinformatics.babraham.ac.uk/projects/fastqc/). All the fastqc files were filtered to remove low-quality reads and adapters with Trimmomatic v.0.3671. The resulting reads were mapped to the *Mus musculus* genome (GRCm38) with HISAT2 v.2.1.072 using default parameters, while Stringtie v1.3.4d73 was applied to the BAM files obtained with HISAT2 to generate expression estimates and to quantify the transcript abundance as transcripts per kilobase per million of mapped reads (TPM). The count matrices generated by Stringtie were imported in R, where differential expression analysis was performed using the Deseq2 package to compare the two different conditions. The functional annotation was performed through the AnnotationDbi R library (http://bioconductor.org/packages/release/bioc/html/AnnotationDbi.html). Functional enrichment analysis was performed by Funrich v3.0 tool (http://funrich.org/index.html) and David 6.8. Data from gene expression omnibus (GEO) were analyzed by GEO2R and integrated by Venn diagram.

### 2.7 Real-time PCR

Total RNA was extracted using TRI Reagent® (Sigma-Aldrich). RNA (3 μg) was retro-transcripted by using M-MLV (Promega, Madison, WI). qPCR was performed in triplicate by using validated qPCR primers (BLAST), Applied Biosystems™ Power™ SYBR™ Green Master Mix, and the QuantStudio3 Real-Time PCR System (Thermo Fisher, Whaltam, MA, USA). mRNA levels were normalized to Rpl8 mRNA, and the relative mRNA levels were determined through the 2−ΔΔCt method.

The nucleotide sequences of the primers employed for mouse samples in the qPCR were as follows:

*Tnfa*: forward, 5′-ATGGCCTCCTCATCAGTT C-3′, reverse, 5′-TTGGTTTGCTACGACGTG-3′;

*Il1b*: forward 5′-AAGTTGACGGACCCCAAAAGAT-3′, reverse 5′-TGTTGATGTGCTGCTGCG A-3′;

*Il6*: forward 5′-AGTTGCCTTCTTGGGACTGA-3′, reverse 5′-TCCACGATTTCCCAGAGAAC-3′;

*Il10*: forward 5′-CGGTACTTGAAGGGCAAAGA-3′, reverse 5′-AACTCCACCTCCTCCAGG TT-3′;

*Ccl2*: forward 5′-TCAGCCAGATGCAGTTAA CG-3′, reverse 5′-GATCCTCTTGTAGCTCTCCAGC-3′;

*Gapdh*: forward 5′-TGGGCTACACTGAGCACCAG-3′, reverse 5′-GGGTGTCGCTGTTGAAGTCA-3′;

*Rpl8*: forward, 5′-GGAGCGACACGGCTACATTA -3′, reverse, 5′-CCGATATTCAGCTGGGCCTT -3′;

*Arg1*: forward, 5′-GGAACCCAGAGAGAGCATGAG -3′,reverse, 5′-CTCGAGGCTGTCCTTTTGAGA -3′;

*Nos2*: forward, 5′-GCCTTCAACACCAAGGTTGTC -3′,reverse, 5′-ACCACCAGCAGTAGTTGCTC -3′;

*Itgam*: forward, 5′-AAACCACAGTCCCGCAGAGA -3′,reverse, 5′-CGTGTTCACCAGCTGGCTTA-3′;

*Adgre*: forward, 5′-TTGGCCAAGATTCTCTTCCT -3′,reverse, 5′-TCACTGCCTCCACTAGCATC -3′;

### 2.8 Analysis of mitochondrial ROS production and lipid peroxidation

Differentiated 3T3-L1 cells were treated with TNFα and DMF and after 24 h were stained using MitoSOX probe (ThermoFisher Waltarm, MA, USA) for 30′ at 37 °C. For Lipid peroxidation analysis, differentiated 3T3-L1 cells downregulated for LCN2 and treated with TNF for 24 h were stained with BODIPY C11 (ThermoFisher, Waltarm, MA, USA) for 30’ at 37 °C. Flow cytometry analysis was performed using Amnis® CellStream® flow cytometry (Luminex company). All Flow cytometry analyses were performed with Amnis® CellStream® software program (Luminex Company).

### 2.9 Fluoromyelin staining

The spinal cord sections were rehydrated in PBS before being incubated with fluoromyelin green fluorescent myelin stain (1:300; Invitrogen, Eugene, OR) for 20 min at 25 °C. The tissues were then washed three times in PBS for 10 min each. Images were obtained under fluorescent microscope and Image J was used to highlight the demyelination area and total white matter area in the spinal cord sections. The pixel area of each sample was calculated, and the percentage of demyelination was measured by dividing the total white matter area by the total demyelinated area.

### 2.10 Immunophenotyping and flow cytometry

Cellular phenotypes were assessed using multiparametric flow cytometry panels containing markers to identify cell types and markers to assess activation states. The use of these markers allowed us to exclude all cells of no interest based on physical parameters (side and forward scatter) and to gate on specific cells of interest. For the immunophenotyping of sWAT, single suspension cells isolated from sWAT were stained with different panels of cell surface markers (see Table 1). For the identification of the main infiltrated leukocyte populations, total leukocytes were identified gating on CD45+ cells. Inside this gate, neutrophils were identified as Ly6G + Ly6Clow cells and monocytes as Ly6C + Ly6Glow cells. Ly6C-Ly6G-cells were further gated to identify CD3+ T-lymphocytes and NK.1+ NK cells. Further gating on CD3-NK.1- cells, allowed us to identify CD19+ B-lymphocytes. In another panel, cells were first gated on CD45+ cells and then on CD11bhighF4/80high to identify total antigen presenting cells, that could be further subdivided into dendritic cells (CD11c + F4/80-) and macrophages (F4/80highCD11c-). Macrophages were further stained for the expression of M1 (anti-CD86, anti-CD40 and anti-MHC-II) or M2 (anti-CD206 and anti-CD200R) markers. Samples were acquired on a 13-color Cytoflex (Beckman Coulter) and for each analysis, at least 0.5 × 10^6^ live cells were acquired by gating on aqua Live/Dead negative cells [35,36]. For the immunophenotyping of spinal cord in adipo-Lcn2 EAE mice, after identifying as CD45 + CD3-population, B-lymphocytes were identified as CD19+ cells and neutrophils as CD11b  +  Ly6G + cells. CD11b  +  Ly6G-cells were further analysed as monocytes as CD19-CD11c-cells and macrophages as F4/80highCD11c-cells. In another panel, CD3+ T-lymphocytes were divided into CD4+ and CD8+ subpopulations. The same gating strategy was applied by staining the single cell suspension with the panel of antibodies shown in Table 2 (Yu Wang). Samples were then acquired on an 18-color LSR Fortessa (Beckton Dickinson). All Flow cytometry analyses were performed with FlowJo software program (Treestar, Ashland, OR, USA).

**Table 1:** Antibodies used for the immunophenotypic characterization of infiltrated immune cell populations within the sWAT.

Antibody	Manufacturer	Dilution
CD4 PerCP5.5	Biolegend	1:100
Ly6G FITC	Biolegend	1:100
CD3 PE	Biolegend	1:100
NK.1. APC	Miltenyi biotec	1:100
Ly6C PE-Cy7	Biolegend	1:100
CD19 APC-Cy7	Biolegend	1:100
F4/80 APC	Biolegend	1:100
CD11c PE-Cy7	Biolegend	1:80
CD11b APC-Vio770	Miltenyi biotec	1:80
CD86 FITC	Miltenyi biotec	1:100
CD40 PE	Miltenyi biotec	1:80
MHC-II PE-Vio770	Miltenyi biotec	1:150
CD206 PE-Cy7	Biolegend	1:100
CD200R PE	Miltenyi biotec	1:100

**Table 2:** Antibodies used for the immunophenotypic characterization of infiltrated immune cell populations within the spinal cord.

Antibody	Manufacturer	Dilution
CD45 Pacific Blue	Biolegend	1:100
Ly6G BV711	Biolegend	1:100
CD3 FITC	Biolegend	1:100
CD11 b PE/Cyanine5	Biolegend	1:100
CD19 APC	BD Biosciences	1:100
F4/80 APC/Cyanine7	Biolegend	1:100
CD11c PE/Cyanine7	Biolegend	1:100
CD3 Pacific Blue	Biolegend	1:100
CD8a Alexa Fluor 647	Biolegend	1:100
CD4 PE	BD Biosciences	1:100

### 2.11 Spinal cord dissociation and flow cytometry

After isolation of the lumbar spinal canal, the spinal cord sample was cut into small pieces and transferred to a shaking bath at 37 °C in Dulbecco's Modiﬁed Eagle Medium (DMEM) containing 1 mg/mL collagenase Type I (Gibico TM, Waltham, MA, USA). The suspension was then filtered (100 μM) and aspired the supernatant after centrifuging at 500×*g*. The pellet was resuspended in iced phosphate-buffered saline (PBS) and labelled with fluorophore-conjugated antibodies (Table 2). After removing the antibodies, the cell pellet was resuspended in PBS (within 5 nM EDTA) for subsequent analyses.

### 2.12 TNFα ELISA kit

Whole blood samples were collected 4 h after fasting to normalized biochemical parameters. Serum from whole blood was collected and the concentrations of TNFα (R&D SYSTEM, Minneapolis, MN, US), was measured by enzyme linked immunosorbent assay (ELISA) kit according to the Manufacturer's protocols.

### 2.13 Statistical analysis

Data are reported as mean ± SD. Statistical analyses were performed using GraphPad Prism software version 9.5.0. Differences between groups which involved a single variable or factor were analyzed using Student T test or a one-way analysis of variance (ANOVA). In groups involving two or more factors, a two-way ANOVA test was performed. All analyses were subjected to Tukey's correction for multiple comparisons and statistical significance was accepted at p < 0.05.

## 3 Results

### 3.1 EAE induces at loss and resident macrophage activation

**Figure 1:**
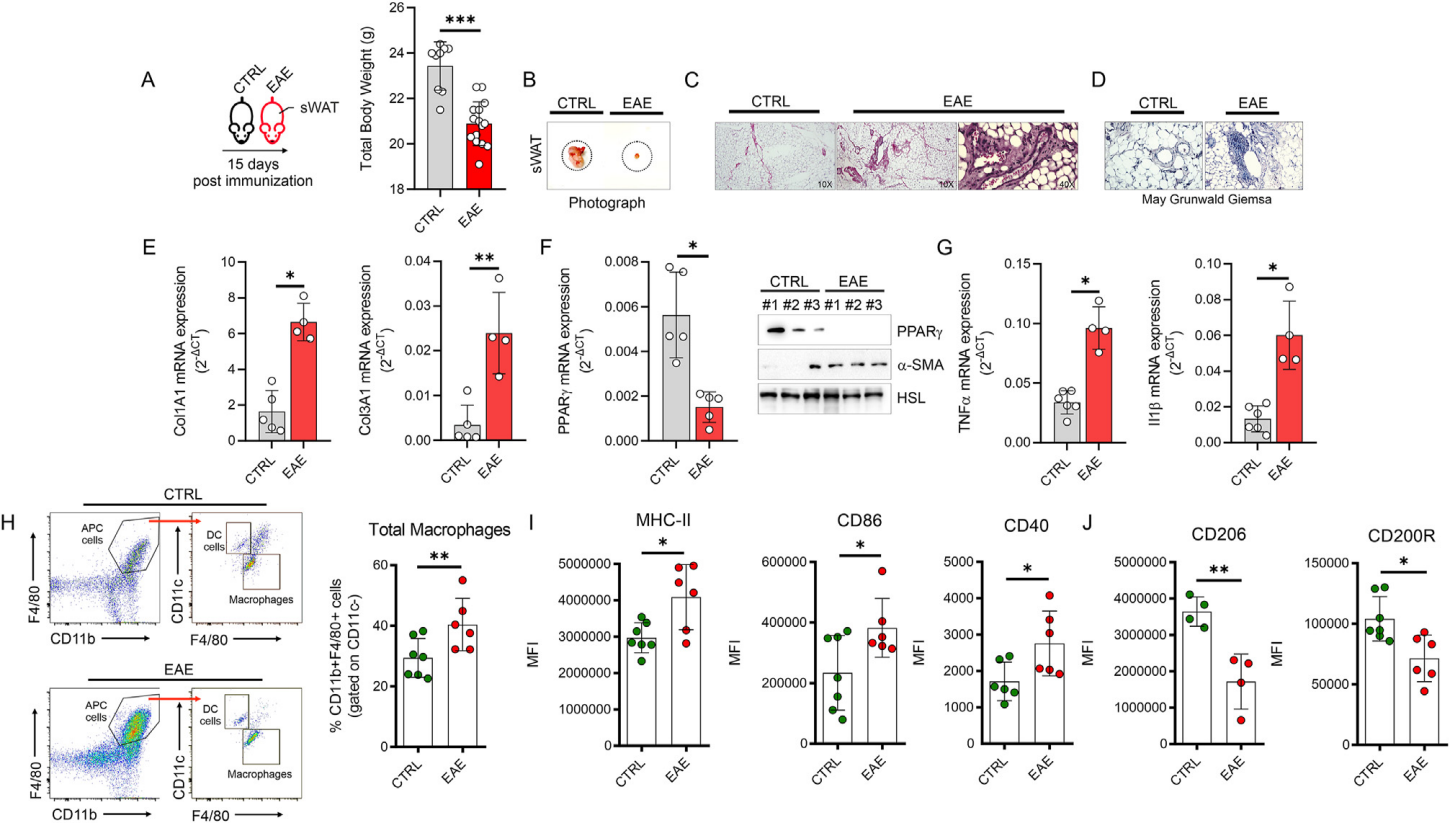
**EAE immunization induces AT loss, inflammation and fibrosis.** (A) Body weight was measured at 15 days post EAE induction (n = 9 Ctrl and n = 15 EAE mice). (B–D) Representative photograph (B), hematoxylin/eosin (C) and May Grunwald Giemsa (D) in sWAT of Ctrl and EAE mice 15 days post immunization (n = 5 Ctrl and n = 5 EAE mice). (E) Col1A1, Col3A1 and Pparγ mRNA expression in sWAT of Ctrl and EAE mice 15 days post immunization (n = 5 Ctrl and n = 5 EAE mice). (F) Representative immunoblots of α-SMA and PPARγ proteins in sWAT of Ctrl and EAE mice 15 days post immunization (n = 5 Ctrl and n = 5 EAE mice). HSL was used as loading control. (G) Tnfα and Il1β mRNA expression in sWAT of Ctrl and EAE mice 15 days post immunization (n = 6 Ctrl and n = 4 EAE mice). (H–J) Flow cytometry plots of total macrophages (CD45^high^F4/80^+^CD11b^+^CD11c^−^) (H), M1-like macrophages (I) and M2-like macrophages (J) in sWAT of CTRL and EAE mice 15 days post immunization (n = 6 Ctrl and n = 6 EAE mice). Data was reported as mean percentages of positive cells or mean fluorescence intensities ± SD. Student's T test ∗p < 0.05; ∗∗p < 0.01; ∗∗∗p < 0.001 EAE vs Ctrl.

Inflammatory status is a hallmark of multiple sclerosis (MS), a frequent autoimmune demyelinating disease of the central nervous system [37]. Although experimental and clinical data postulated a pathogenic link between dysfunctional AT and MS, the responses of AT during demyelinating conditions have never been explored. In the experimental autoimmune encephalomyelitis (EAE), the most commonly used experimental murine model for the study of MS, ∼15–20% body weight loss (mainly due to the loss of adipose mass) was observed about 15-days post immunization: (15dpi) [[4], [5], [6], [7]]. Here we aimed at investigating the molecular and metabolic features of AT in EAE mice at the onset of disease (15dpi) and in line with other data, a significant loss of the total body weight was recorded (Figure 1A). Next, we asked whether the reduction in the total body weight was caused by reduced food intake. To this end, food consumption was measured every 3 days up to 21-days post immunization and no differences between EAE and CTRL mice were observed (Suppl. Figure 1A). This result led us to postulate that other factors mediated the acute loss in the body weight independently to calorie consumption. In order to investigate the tissue-specific importance underlying observed body weight loss in EAE mice, we analyzed the responses of skeletal muscle and white adipose tissues. Although, skeletal muscle mass (*tiabialis anterior*) was reduced (Suppl. Figure 1B), any modulation in the atrophy-related genes such Atrogin1 and Murf1 was observed in EAE mice (Suppl. Figure 1C). Differently, epididymal white adipose tissue (eWAT) was totally lost in EAE mice at 15 dpi, whereas subcutaneous white adipose tissue (sWAT) mass was strongly reduced (Figure 1B), suggesting that the significant reduction in body weight was mainly attributable to the reduction in the white adipose tissue. Based on these results, we aimed to study the metabolic and molecular signatures of subcutaneous WAT (sWAT). The histological features of sWAT showed massive fibrosis in EAE mice (Figure 1C,D), which was associated with an increased level of fibrosis markers such as *Col1A*, *Col3A1* mRNAs and α-SMA protein (Figure 1E,F). In line with this, diminished levels of anti-fibrotic and pro-adipogenic marker PPARγ were observed in sWAT of EAE mice (Figure 1F). Fibrosis is often associated with unresolved inflammation and persistence of inflammatory stress [38]. Accordingly, increased expression of inflammatory genes such as *Tnfα* and *Il1β* was observed in sWAT of EAE mice (Figure 1G), which was consistent with the increased circulatory levels of TNFα (Suppl. Figure 1D). Next, to better clarify the immune cells in the sWAT of EAE mice, we performed a immunophenotyping through high dimensional flow cytometry (Suppl. Figure. 1E and Figure 1H). A first analysis of the total CD45^+^ cells revealed that leukocytes were significantly increased in sWAT of EAE (Suppl. Figure 1E). When dissecting the individual contribution of the different leukocyte populations, we found that CD19^+^ B-lymphocytes, CD3^+^ T-lymphocytes, CD11c^+^CD11b^+^Ly6C^−^ dendritic cells and Ly6G^+^Ly6C^−^ neutrophils remained unchanged (Suppl. Figure 1F), whereas NK1.1^+^ NK cells, Ly6C^+^ monocytes and F4/80^+^CD11b^+^CD11c^−^ total macrophages were increased in sWAT of EAE mice (Suppl. Figure 1G and Figure 1H). To further characterize macrophage inflammatory activity and polarization, we assessed the expression of key M1-like and M2-like markers and we found that sWAT of EAE mice displayed an accumulation of M1-like macrophages (Figure 1I), characterized by higher levels of MHC-II, CD86 and CD40, and a concomitant reduction of CD206-and CD200R-expressing M2-like macrophages (Figure 1J) was observed, suggesting that innate immunity, and in particular monocytes/macrophages, promotes an inflammatory milieu surrounding adipocytes.

### 3.2 LCN2 is a hallmark of dysfunctional AT

**Figure 2:**
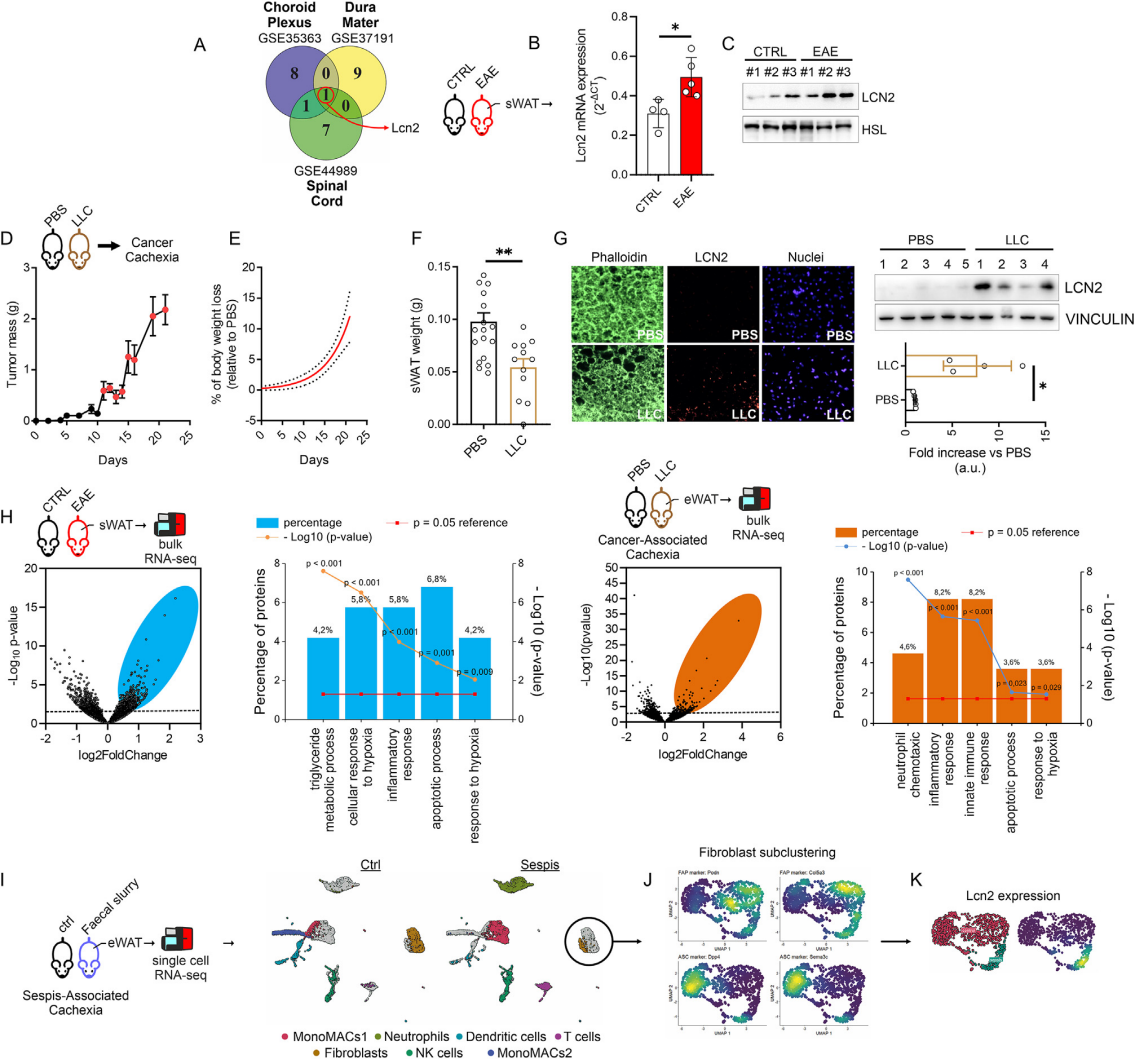
**LCN2 is induced in AT of EAE, cancer- and sepsis-inducing cachectic mice.** (A) Venn diagram including the up-regulated genes in choroid plexus, dura mater and spinal cord of EAE mice. (B, C) Lcn2 mRNA (B) and protein (C) expression. HSL was used as loading control. HSL was the same as shown in Figure 1F because it is part of the same western blot. (D, E) Tumor mass progression (D), % body weight loss (E) in mice during LLC post inoculation (n = 17 PBS and n = 11 LLC mice). (F) sWAT weight 21 days post inoculation (n = 17 PBS and n = 11 LLC mice). Representative immunofluorescence of Phalloidin (green), LCN2 (red), Dapi (blue) in sWAT of PBS and LLC mice 21 days post inoculation (n = 5 PBS and n = 4 LLC mice). Representative immunoblots of LCN2 in in sWAT of PBS and LLC mice 21 days post inoculation (n = 5 PBS and n = 4 LLC mice). VINCULIN was used as loading control. (H) Volcano plot and functional enrichment analysis for biological processes of up-regulated genes (Log2FC > 1.5; pAdj<0.05) in sWAT of EAE and LCC mice (n = 5 mice/group). (H) Single cell RNA-sequencing (PRJNA626597) of visceral white adipose tissue isolated from mice 1 month after sepsis induction. (J) Fibroblast annotation for fibroblast adipocyte precursors (FAP: Podh, Col5a3) and adipose stem cells (ASC: Dpp4, Sema3c). (K) Lcn2 expression in fibroblast of control and sepsis mice. Data was reported as mean ± SD. Student's T test ∗p < 0.05; ∗∗p < 0.01; ∗∗∗p < 0.001 treated vs Ctrl.

To identify molecular mediators linking AT remodeling and immunological perturbations in MS, we initially applied a text-data mining strategy by analyzing the top 10 up-regulated genes (p_adj_ < 0.05) in the spinal cord (GSE44989), dura mater (GSE37191) and choroid plexus (GSE35363) isolated from EAE mice. Through Venn diagram we identified *Lcn2* as the unique overlapping gene (Figure 2A), suggesting its predictive role in the MS pathogenesis. Interestingly, a high expression level of *Lcn2* was observed in sWAT at the onset of EAE (Figure 2B,C), leading us to suppose that its induction accompanies the pathological loss of AT. To give more insight on this aspect, we generated an *in vivo* model of cancer cachexia by inoculating Lewis Lung Carcinoma cells (LCC) in wild type mice. Body weight and tumor mass were monitored every other day up to 21-days post injection. Tumor mass weight increased progressively with a doubling time of 3.8 g/day (Figure 2D) starting from day 11 dpi. In parallel we observed a cachectic index (CI), expressed as percentage of body weight loss, which reached approximately 11% of increase 21 dpi (Figure 2E). The decrease in the body weight was not associated with reduced food intake (Suppl. Figure 2A), whereas a significant reduction of sWAT (Figure 2F) and a substantial increase of LCN2 protein level was observed (Figure 2G). Similar results were observed in eWAT, in which LCN2 protein levels were increased concomitantly to eWAT mass loss (Suppl. Figure 2B). To corroborate the occurrence of cachectic phenotype, skeletal muscle mass and markers of muscle atrophy such as Atrogin 1 and Murf1 were measured. As reported in Suppl. Figure 2C, although the *tibialis anterior* weight was unaffected in LLC mice, an increased expression level of Atrogin1 was observed. To explore the mechanisms governing *Lcn2* up-regulation in cachectic AT, we performed a bulk RNA-sequencing in both EAE and LCC mice. The up-regulated genes (Log2FC > 0.6; p < 0.05) were analyzed for biological processes (Figure 2H) and among the main representative processes, we observed inflammatory and hypoxia responses in both conditions (Figure 2H). In order to broaden our evidence, we aimed at analyzing the cellular landscapes of AT following sepsis-induced cachexia (SIC), which causes AT loss similarly to EAE and LCC mice [39]. Consistent with cancer cachexia and EAE mouse models, an increase in macrophages as well as neutrophils and T cells was observed in AT of SIC mice (Figure 2I). Through MacSpectrum tool we analyzed macrophage polarization index (MPI) and the activation-induced macrophage differentiation index (AMDI) [40], and we identified that AT-resident macrophages in SIC mice developed M1-like phenotype (Suppl. Figure 2D). Consistently, Lcn2 expression was abundant in neutrophils and in a subpopulation of fibroblast (Suppl. Figure 2E). To explore if Lcn2 was induced in adipocyte precursors following sepsis condition, we subclusterized fibroblasts (Figure 2J) and a significant increase of Lcn2 expression was detected in fibroblast adipocyte precursors (FAP) (Figure 2K). Based on these data, we asked if Lcn2 induction in FAP was part of an adaptive inflammatory response. To solve this question, we performed a gene set enrichment analysis and an increased inflammatory pathway was observed in sepsis (Suppl. Figure 2F). Overall collected data suggest that adipocyte-related *Lcn2* participates as stress responsive protein during inflammatory conditions associated with cachexia-like states.

### 3.3 TNFα-treated adipocytes release LCN2 through redox-dependent mechanism

**Figure 3:**
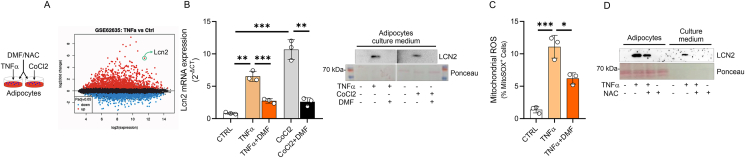
**Inflammatory or hypoxic stimuli induce Lcn2 expression through redox-dependent mechanism.** (A) Differentially expressed genes in mature 3T3-L1 adipocytes treated with TNFα (GSE62635). (B) Lcn2 mRNA and protein expression in TNFα- (10 ng/mL: 6 h in serum-free media), CoCl2-treated (0.5 mM: 6 h in serum-free media) or untreated mature adipocytes. Mature adipocytes were preconditioned with DMF (50 μM) for 24 h. Ponceau staining was used as loading control. (C) Mature 3T3-L1 adipocytes were labelled with MitoSox for 30 min and then stimulated with TNFα (10 ng/mL: 6 h in serum-free media). DMF (50 μM) was added 24 h prior TNFα stimulation. (D) Representative immunoblots of LCN2 in cell culture medium. Mature 3T3-L1 adipocytes treated with TNFα (10 ng/mL: 6 h in serum-free media) and N-acetyl cysteine (NAC: 2 mM) was added 1-hour prior TNFα stimulation. Ponceau staining was used as loading control. Data was reported as mean ± SD. ANOVA test ∗p < 0.05; ∗∗p < 0.01; ∗∗∗p < 0.001.

Based on transcriptomics data of AT in EAE and cachectic mice, herein we postulated that LCN2 induction was consequential to inflammatory or hypoxic conditions in adipocytes. To test this, mature adipocytes were treated TNFα or cobalt chloride (CoCl_2_), a chemical model to induce hypoxia *in vitro* [41,42]. Interestingly, both TNFα and CoCl_2_ caused LCN2 release from adipose cells (Figure 3A,B) as well as mitochondrial reactive oxygen species (ROS) production (Figure 3C), which is consistent with the effectiveness of TNFα to promote mitochondrial damage in adipocytes ([43]{Chen, 2010 #59)}. To give more insight about the redox control of LCN2 induction in dysfunctional adipocytes, we aimed at using dimethylfumarate (DMF), a fumaric acid ester approved for the treatment of relapsing-remitting MS, which promote Nrf2-mediated antioxidant defense [44,45]. As expected, DMF reduced the expression and release of LCN2 in TNFα- and CoCl_2_-treated 3T3-L1 white adipocytes (Figure 3A,B) and this response was accompanied by nuclear accumulation of Nrf2 (Suppl. Figure 2G). In accordance with the antioxidant role of Nrf2 [46], we found that DMF increased the expression levels of antioxidant genes (Suppl. Figure 2H) and diminished the production of mitochondrial ROS in TNFα-treated adipocytes (Figure 3C). Remarkably, treatment with the antioxidant N-acetylcysteine (NAC) prevented LCN2 induction and release from TNFα and CoCl2-treated adipocytes (Figure 3D and Suppl. Figure 2I). Next, we selectively induced mitochondrial stress by using an inhibitor of electron transport chain such as Antimycin A (AA). As expected, a significant increase of LCN2 was observed in mature adipocytes, which was effectively prevented by NAC (Suppl. Figure 2J). The collected results suggest that LCN2 take part to mitochondrial stress response in adipose cells.

### 3.4 Adipose Lcn2 controls TNFα-mediated body weight loss and macrophages activation

**Figure 4:**
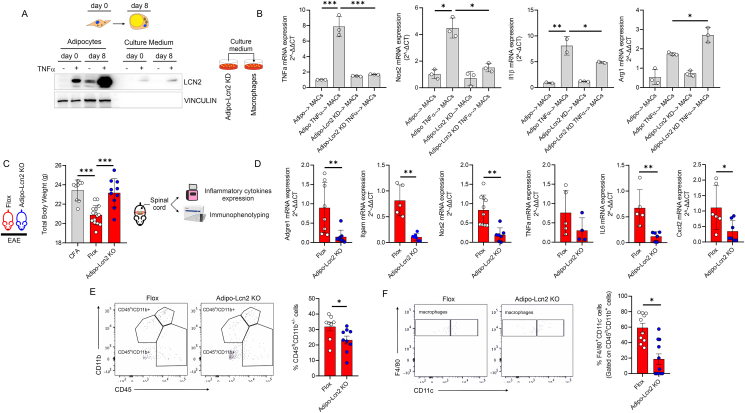
**Adipose-derived LCN2 promote macrophage inflammatory activation.** (A) Representative immunoblots of LCN2 in undifferentiated (day 0) and fully differentiated (day 8) cells and culture medium. VINCULIN was used as loading control. (B) TNFα, Nos2, Il1β and Arg1 mRNA expression in RAW264.7 co-cultured with adipocytes downregulating LCN2 (Lcn2 KD). TNFα (10 ng/mL: 6 h in serum-free media) was added to co-culture system. (C) Total body weigh 15-day post immunization in Flox and Lcn2 KO mice specifically in adipocytes (Adipo-Lcn2 KO) (n = 9/15 mice/group). (D) Gene expression markers of macrophages in the spinal cord of flox and Lcn2 KO mice specifically in adipocytes (Adipo-Lcn2 KO). (E, F) Flow cytometry plots of infiltrated leukocytes (CD45^high^CD11b^+/−^) and macrophages (CD45^high^F4/80^+^CD11b^+^CD11c^−^) in the spinal cord of Flox and Lcn2 KO mice specifically in adipocytes (Adipo-Lcn2 KO) (n = 8/9 mice/group). Data was reported as mean percentages of positive cells ± SD. Student's T test or ANOVA test ∗p < 0.05; ∗∗p < 0.01; ∗∗∗p < 0.001.

**Figure 5:**
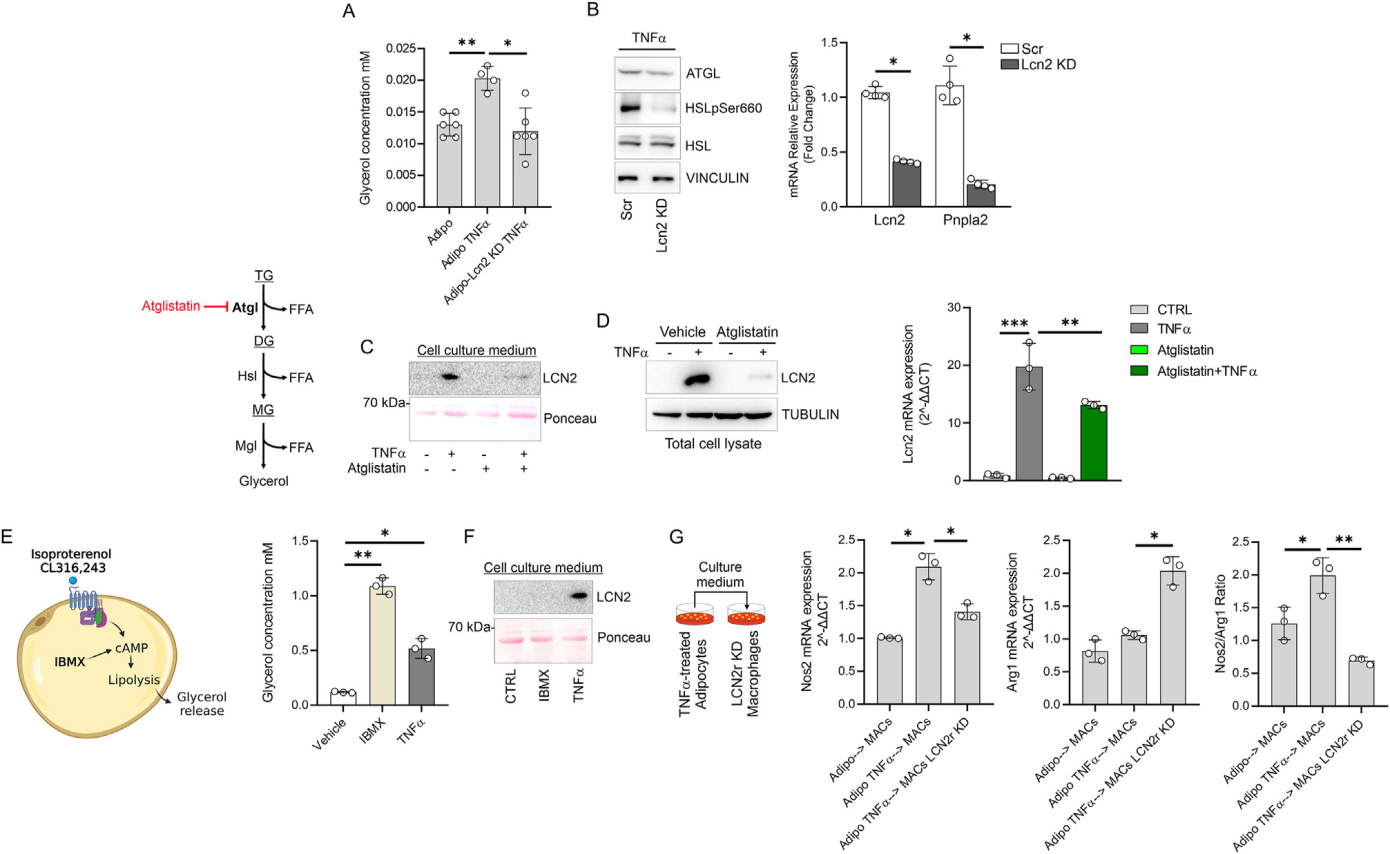
**LCN2 participates in the uncanonical lipolysis of adipocytes.** (A) Representative immunoblots of ATGL, HSLpSer660 in adipocytes downregulating LCN2 (Lcn2 KD) treated with TNFα (10 ng/mL: 6 h in serum-free media) (*left panel*). HSL and VINCULIN were used as loading controls. Lcn2 mRNA expression in adipocytes downregulating LCN2 (Lcn2 KD) treated with TNFα (10 ng/mL: 6 h in serum-free media) (*right panel*). (B) Glycerol release from adipocytes downregulating LCN2 (Lcn2 KD) treated with TNFα (10 ng/mL: 6 h in serum-free media). (C) Representative immunoblots of LCN2 in cell culture medium of adipocytes treated with TNFα (10 ng/mL: 6 h in serum-free media). Atglistatin (200 μM) was added 1 h prior to TNFα treatment. Ponceau staining was used as loading control. (D) Representative immunoblots of LCN2 (*left panel*) and mRNA expression (*right panel*) in adipocytes treated with TNFα (10 ng/mL: 6 h in serum-free media). Atglistatin (200 μM) was added 1 h prior to TNFα treatment. TUBULIN was used as loading control. (E) Glycerol release from adipocytes treated with IBMX (500 μM) or TNFα (10 ng/mL) for 8 h. (F) Representative immunoblots of LCN2 in cell culture medium of 3T3-L1 adipocytes treated with IBMX (500 μM) or TNFα (10 ng/mL) for 8 h. Ponceau staining was used as loading control. (G) Nos2, Arg1 mRNA expression and Nos2/Arg1 mRNA ratio in RAW264.7 downregulating LCN2 receptor (LCN2r KD) co-cultured with TNFα-treated adipocytes. TNFα (10 ng/mL: 6 h in serum-free media) was added to co-culture system. Data was reported as mean percentages of positive cells ± SD. Student's T test or ANOVA test ∗p < 0.05; ∗∗p < 0.01.

LCN2 is a protein that is particularly represented in the adipose tissue (also called as adipokine) and its global depletion protects from demyelination (Suppl. Figure 3A) and neuroinflammatory setting in the spinal cord of EAE mice (Suppl. Figure 3B). Accordingly, Lcn2 treatment activated microglial cells to levels comparable to TNFα (Suppl. Figure 3C), suggesting a key role of LCN2 in driving inflammation. Remarkably, the transcriptome of female mice overexpressing LCN2 specifically in the adipose depots (Adipose^LCN2up^) showed an increased expression of *Hif1a* as well as of several fibrosis and inflammatory markers including *Col6a1*, *Col5a2*, *Col6a3*, *Ccl2*, *S100a8*, *Saa3* [47]. Overall, these data suggest that adipose-derived LCN2 could be part of an autoregulatory loop linking adipocyte dysfunction to macrophage activation. To explore the role of adipose-derived LCN2 in macrophage activation, we tested whether LCN2 was peculiar to mature adipocytes. To this end, we compared the levels of LCN2 in differentiated (day 8) versus undifferentiated (day 0) adipocytes and higher release of LCN2 was detected in mature adipocytes (Figure 4A). Next, we carried out TNFα treatment on adipo-Lcn2 KD and the culture media were collected and used to treat macrophages. The analysis of inflammatory cytokines expression showed that macrophages cultured with adipo-Lcn2 KD and TNFα-treated conditioned medium showed reduced expression of pro-inflammatory M1-like markers such as TNFα, Nos2 and IL1β and concomitant higher levels of anti-inflammatory M2-like marker Arg1 with respect to macrophages cultured with conditioned medium from TNFα-treated adipocytes with normal levels of LCN2 (Figure 4B). These data corroborate the inflammatory role of adipose-derived LCN2 and draw a direct link between this adipokine, AT loss and M1-like macrophage activation. Consistent with this, was recently demonstrated that Lcn2 downregulation, limited AT loss and inflammatory cell infiltration in a pancreatic cancer cachexia mouse model [21]. Based on these data, we aimed at exploring the role of adipose-derived LCN2 in EAE mice. To this end a mouse model where LCN2 is specifically downregulated in the adipose tissue (adipo-*Lcn2* KO) was generated and the inflammatory phenotype of EAE mice was analyzed. Although we did not observe any improvement in the clinical score (data not shown), adipo-*Lcn2* KO were resistant to body weight loss following EAE induction compared to control mice (CFA) (Figure 4C). Interestingly, the spinal cord of adipo-*Lcn2* KO revealed a diminished mRNA expression of monocytes/macrophages markers such as Adgre1 (F4/80), Itgam and Nos2 (Figure 4D), as well as inflammatory cytokines and chemokines such as *Tnfa*, *Il6 and Cxcl2* (Figure 4D) compared to adipo-*Lcn2* Flox. In line with these data, immunophenotyping of leukocytes within the spinal cord through high dimensional flow cytometry revealed a diminished number of total infiltrated leukocytes (CD45^high^CD11b^+/−^) (Figure 4E) and macrophages (CD45^high^F4/80^+^CD11b^+^CD11c^−^) (Figure 4F). Consistent with recent findings (das Neves et al., 2022), our results suggest that adipose-LCN2 participates in the AT loss and exacerbates the innate immune response during inflammatory conditions. To explore the role of LCN2 in non-hormonally-stimulated adipocytes, we downregulated LCN2 specifically in mature adipocytes (adipo-*Lcn2* KD) and lipolysis markers were analyzed following TNFα treatment. Interestingly, adipo-*Lcn2* KD showed a limited glycerol release from adipo-*Lcn2* KD (Figure 5A). Similar finding was observed in AT of cachectic mice [21], suggesting a key role of LCN2 in mediating AT loss during cachectic-like conditions. Remarkably, DMF efficiently reduced glycerol release from TNFα and CoCl_2_-treated adipocytes (Suppl. Figure 3D). The significant reduction in the glycerol release was accompanied with the reduced levels of the rate limiting enzyme of lipolysis ATGL (adipose triglyceride lipase) and the phospho-active form of HSL (hormone sensitive lipase) (Figure 5B). Recent findings revealed that ATGL inhibition in AT, rather than other lipases, protects from tumor-associated AT cachexia [48]. To demonstrate if ATGL also controls LCN2 induction during cachectic stimuli, we selectively inhibited ATGL by Atglistatin and we observed a limited induction and release of LCN2 from TNFα-treated adipocytes (Figure 5C,D). Next, we asked whether the mechanism promoting LCN2 release by TNFα belongs to the canonical lipolysis signaling pathways. To test this, mature 3T3-L1 adipocytes were treated with 3-isobutil-1-metilxantina (IBMX), a cell-permeable inhibitor of cAMP phosphodiesterases, which activates cAMP-dependent protein kinase (cAMP/PKA). Although IBMX massively increased glycerol release from mature adipocytes (Figure 5E), LCN2 expression and release were unffected (Figure 5F). Remarkably, lipolysis activation by β3-receptor agonist isoproterenol increased glycerol release (Suppl. Figure 4A), whereas LCN2 induction and release LCN2 were unaffected (Suppl. Figure 4B). The collected results suggest that LCN2 released from adipose cells is part of an adaptive stress response to inflammatory mileua. Consistent with this, similar to TNFα, also IL6 strongly increased LCN2 induction and release from adipocytes (Suppl. Figure 4C).

The reciprocal relationship between LCN2 manipulation and lipolysis makes it difficult to interpret the release of LCN2 as the driver of macrophage inflammation. Accordingly, several authors demonstrated that free fatty acids released from dysfunctional adipocytes elicit macrophage inflammation [49]. To demonstrate a direct role of adipose-derived LCN2 to promote the inflammatory responses in macrophages, we co-cultured TNFα-treated adipocytes with macrophages downregulating LCN2 receptor (LCN2r KD). As reported in Figure 5G, macrophages LCN2r KD showed a limited inflammatory activation when co-cultured with dysfunctional adipocytes. These data demonstrate that the inflammatory inputs are causative of LCN2 release from adipose cells, which directly activates macrophages through LCN2 receptor.

## 4 Discussion

Adipose tissue (AT), a large lymphoid tissue characterized in heterogenous cell population including immune cells [50]. Factors secreted by adipose tissue have an impact on immuno-metabolic homeostasis, contributing to several inflammatory-associated diseases. Interestingly, the treatment with metformin and pioglitazone, two compounds used for the management of metabolic syndrome, has been shown to increase the adiponectin/leptin ratio and improve the inflammatory profile, coinciding with decreased brain lesions in MS patients [51] and reduced cancer risk [52]. Adipocytes also release extracellular vesicles (EVs) that play a role in several pathophysiological conditions [53]. It is worth noting that EVs released by adipocytes stimulate fatty acid oxidation and migration in melanoma cells [54] and are involved in macrophage activation [55]. These findings emphasize the importance of monitoring the AT secretome to enhance disease-modifying therapies for conditions such as multiple sclerosis (MS) [56,57] and cancer cachexia [21,58]. Emerging findings revealed that adipose-derived LCN2 participates in several pathophysiological conditions including inflammation [21,25,26,31,47]. LCN2 is a prognostic marker of cancer-associated cachexia (CAC) and its downregulation improve CAC symptoms. High expression levels of LCN2 were observed in several pre-clinical models of MS as well as in human. *In vitro* studies demonstrated the capability of LCN2 to promote inflammatory cells activation such as microglial and macrophages [59,60]. For the first time, our study explored the responses of AT in EAE mice revealing a cachectic-like condition. We identified LCN2 as stress responsive protein released by dysfunctional adipocytes, which activates innate immunity. LCN2 downregulation improved demyelination and inflammatory status in EAE mice. Of note, when LCN2 was downregulated specifically in AT, EAE mice were protected against body weight loss and inflammatory macrophage infiltration in the spinal cord. However, despite thoroughly characterizing the effects of LCN2 on macrophage inflammation, it appears that animals lacking LCN2 in adipocytes do not show improvement in their clinical score in the EAE model. The rationale behind our findings stems from the profound impact of macrophage-driven innate immunity on the clinical scores of experimental autoimmune encephalomyelitis (EAE), particularly during the chronic phase that occurs at 25–30 dpi. The spatio-temporal distributions and contributions to disease development have revealed that monocytes/macrophages play a role in the early (pre-symptomatic) phase of EAE, promoting the activation of T cell-mediated autoreactivity [61] and the onset of MS-related symptoms. The significant reduction of macrophages in the spinal cord of Adipo-Lcn2 KO mice in the early phase of EAE strongly suggests that their pathogenic activity will subsequently be reflected in an attenuated chronic phase of the disease. Consequently, we anticipate that the influence of adipocyte-derived Lcn2 on the clinical score will become evident during the later phase of the disease.

Similar findings reported that LCN2 downregulation, limited AT loss and tissue macrophage infiltration in a murine model of cancer cachexia [21]. In murine model of renal injury, LCN2 downregulation specifically in adipose depots, protects protected disease progression [31]. In line with these findings, female mice overexpressing LCN2 specifically in AT showed increased fibrosis and tissue inflammation [47].

Emerging findings highlighted a role of LCN2 in lipolysis and lipid peroxides production, which are markers of ferroptosis [62,63]. This is consistent with data reporting that Lcn2 expression is induced by reactive oxygen species and its downregulation protect against from oxidative stress, inflammation and ferroptosis [[63], [64], [65], [66]]. Herein, we demonstrate that the release of LCN2 from adipocytes is strictly dependent on inflammatory milieu. In fact, the canonical stimulation of lipolysis by hormones was ineffective in promoting LCN2 release from adipocytes, thereby highlighting a pathognomonic function of LCN2 in dysfunctional adipocytes. In adipose cells, treatment with the Nrf2-agonist dimethyl fumarate (DMF) effectively reduced LCN2 induction following inflammatory or hypoxic stimuli. Of note DMF prevents weight loss in murine models of MS [67] and sepsis [68]. Although DMF is a first-line-treatment for relapsing-remitting multiple sclerosis (RRMS), the mechanisms of action remain still unclear. Herein we provided evidence that DMF might limit MS-related symptoms through the reduction of LCN2 production from dysfunctional adipocytes.

In conclusion, we identified adipose-derived LCN2 as a stress responsive protein, which participates in several pathological conditions exacerbating inflammatory responses and disease progression. In this scenario, obesity take part to EAE disease progression by releasing higher amount of LCN2 from adipose depots.

## Author contribution

DLB conceived the whole study and designed experiments. DLB and KA jointly supervised this work. FS, VC,VCh designed and performed most of the experiments and analyzed data. VCh supervised high dimensional flow cytometry with the help of MT and AM. ZYZ, QS, DK performed experiments on Adipo-LCN2 KO mice supervised by YW. KS and AB performed experiments on LCN2 KO mice. AN and FZ helped with the analysis of RNA sequencing data. MR and CDB performed experiments on cachectic mice. DF and SB performed EAE immunization supervised by DC. DLB wrote and revised the manuscript. VCh, YW, KS and KA revised the manuscript.

## Declaration of competing interest

The other authors declare no competing interests.

## Figures and Tables

**Figure:**
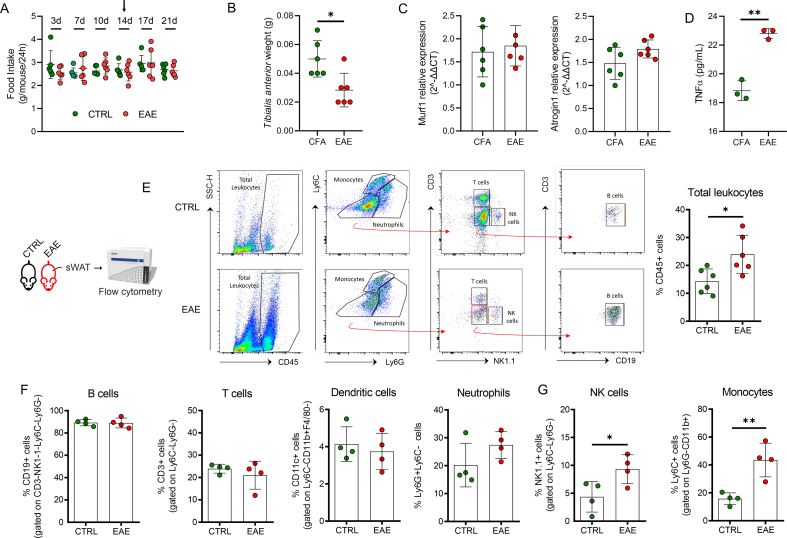


**Figure:**
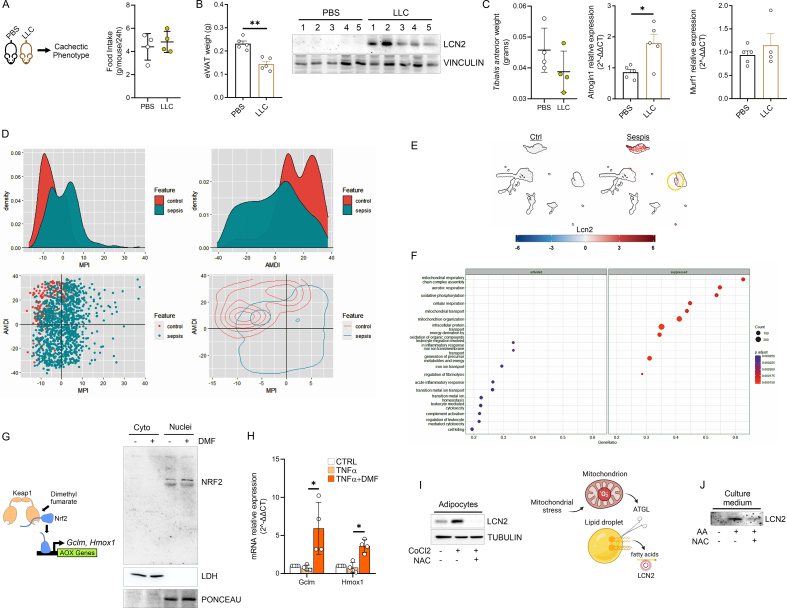


**Figure:**
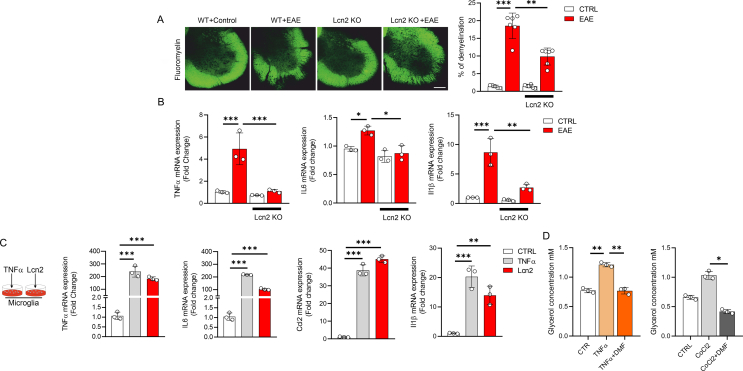


**Figure:**
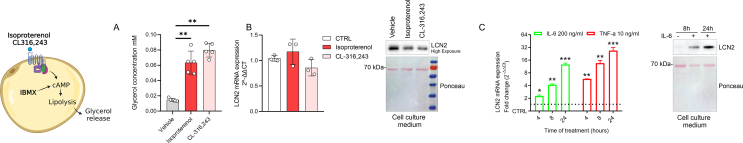


## Data Availability

Data will be made available on request.
